# Diurnal oscillations in human salivary microRNA and microbial transcription: Implications for human health and disease

**DOI:** 10.1371/journal.pone.0198288

**Published:** 2018-07-18

**Authors:** Steven D. Hicks, Neil Khurana, Jeremy Williams, Cindy Dowd Greene, Richard Uhlig, Frank A. Middleton

**Affiliations:** 1 Department of Pediatrics, Penn State University Hershey Medical Center, Hershey, PA, United States of America; 2 Department of Neuroscience & Physiology, SUNY Upstate Medical University, Syracuse, NY, United States of America; 3 Quadrant Biosciences, Inc., Syracuse, NY, United States of America; 4 Department of Psychiatry & Behavioral Sciences, SUNY Upstate Medical University, Syracuse, NY, United States of America; 5 Department of Biochemistry & Molecular Biology, SUNY Upstate Medical University, Syracuse, NY, United States of America; 6 Department of Pediatrics, SUNY Upstate Medical University, Syracuse, NY, United States of America; University of Massachusetts Medical School, UNITED STATES

## Abstract

The microbiome plays a vital role in human health and disease. Interaction between human hosts and the microbiome occurs through a number of mechanisms, including transcriptomic regulation by microRNA (miRNA). In animal models, circadian variations in miRNA and microbiome elements have been described, but patterns of co-expression and potential diurnal interaction in humans have not. We investigated daily oscillations in salivary miRNA and microbial RNA to explore relationships between these components of the gut-brain-axis and their implications in human health. Nine subjects provided 120 saliva samples at designated times, on repeated days. Samples were divided into three sets for exploration and cross-validation. Identification and quantification of host miRNA and microbial RNA was performed using next generation sequencing. Three stages of statistical analyses were used to identify circadian oscillators: 1) a two-way analysis of variance in the first two sample sets identified host miRNAs and microbial RNAs whose abundance varied with collection time (but not day); 2) multivariate modeling identified subsets of these miRNAs and microbial RNAs strongly-associated with collection time, and evaluated their predictive ability in an independent hold-out sample set; 3) regulation of circadian miRNAs and microbial RNAs was explored in data from autistic children with disordered sleep (n = 77), relative to autistic peers with typical sleep (n = 63). Eleven miRNAs and 11 microbial RNAs demonstrated consistent diurnal oscillation across sample sets and accurately predicted collection time in the hold-out set. Associations among five circadian miRNAs and four circadian microbial RNAs were observed. We termed the 11 miRNAs CircaMiRs. These CircaMiRs had 1,127 predicted gene targets, with enrichment for both circadian gene targets and metabolic signaling processes. Four CircaMiRs had “altered” expression patterns among children with disordered sleep. Thus, novel and correlated circadian oscillations in human miRNA and microbial RNA exist and may have distinct implications in human health and disease.

## Introduction

The proper regulation of sleep in humans is critical for normal mental and physical health. Most major organ systems exhibit fluctuations in their functional state related to sleep-wake cycles or circadian rhythm [1–3]. Disturbances in sleep or disruption of circadian rhythm are a common problem in many chronic brain disorders, including autism, depression, Parkinson’s, and Alzheimer’s. These symptoms have a negative impact on activities of daily living [3].

During sleep-wake cycles there are numerous molecular, cellular, and physiological changes that occur. Many of these changes are driven by circadian regulatory genes, such as CLOCK and BMAL [4]. These, in turn, cause a vast array of changes in the expression of physiologically significant genes, proteins, and hormones, influencing nearly every body system. However, apart from light-dark cycles, the factors that influence expression of circadian rhythm are not fully understood.

MicroRNAs (miRNAs) are small, noncoding RNA fragments, approximately 20–22 nucleotides long in their mature state. MiRNAs are involved in post-transcriptional regulation of gene expression [5–8]. After processing by endonucleases [8, 9], single-stranded miRNAs combine with other macromolecules to form RNA-induced silencing complexes (RISCs). RISCs target complementary messenger RNA (mRNA) strands for degradation and interfere with translation, thereby altering cellular function [8, 9]. MiRNAs exert widespread influence on gene expression. More than 1900 identified miRNAs have been shown to affect the expression of up to 60% of all genes [10–13]. MiRNAs play a role in virtually all cellular functions, such as cell proliferation, differentiation, and apoptosis [6, 10, 11].

MiRNAs are found in nearly all body cells, tissues, and biofluids [10, 14]. Because miRNAs regulate the majority of human genes, a considerable number of circadian genes are now thought to be directly under their influence, including CLOCK and BMAL, among others [15]. MiRNAs that circulate throughout the body in extracellular fluids are also resistant to enzymatic degradation [16], and thus may act as critical components of a molecular endocrine system [17]. Indeed, there are now considerable data implicating miRNAs in the control of various endocrine and metabolic tissues, such as the pineal and pituitary glands [18], the hypothalamus, and the gastrointestinal (GI) tract. Furthermore, disruption of circadian regulation by miRNAs can lead to significant pathology [19].

Notably, the activities of miRNAs in the gut appear to extend beyond the regulation of host gene expression, and include a strong relationship with the resident bacteria of the microbiome [20, 21]. Within the GI system, the microbiome contributes to energy harvesting by generating numerous metabolites and intermediates that influence the function of other organ systems, including the brain and endocrine organs [22]. Recent evidence also indicates that there are circadian changes in the gut microbiome [23]. Thus, cross-talk between host miRNAs and the GI microbiome may work in concert to influence temporal changes in gene expression that drive host behavior and disease.

To our knowledge, only one prior study has demonstrated diurnal variations for a select number of cell free microRNAs in human plasma, using quantitative RT-PCR [24]. However, no prior studies have harnessed next-generation sequencing to investigate diurnal variations for the entire micro-transcriptome, or explored these diurnal patterns in the GI tract parallel to the microbiome. We hypothesized that 1) a saliva-based collection method would identify host miRNA and microbial RNA elements with consistent and parallel circadian oscillations; 2) these RNA elements would target functionally-relevant biologic pathways related to host immunity, circadian rhythm, and metabolism; and 3) a subset of circadian miRNAs would demonstrate “altered” expression in a cohort of children with disordered sleep patterns.

## Results

### Salivary miRNA analysis

**Fig 1:**
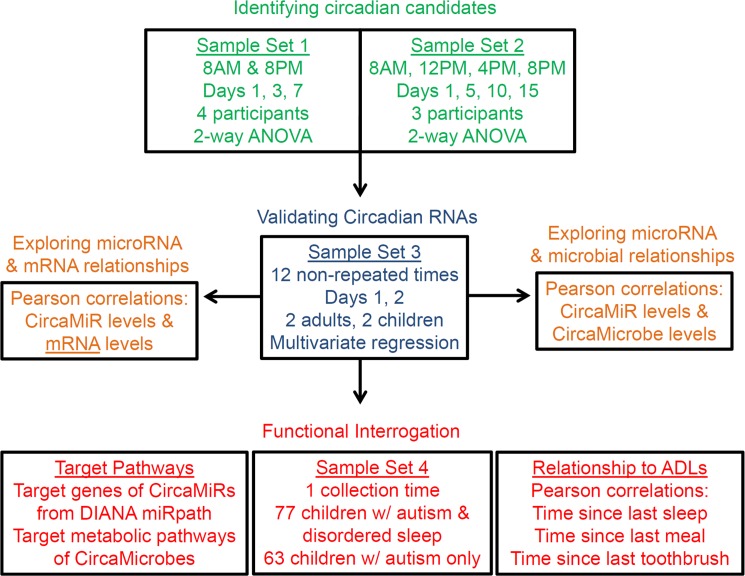
Flow chart outlining the analytic approach for the current study. Briefly, sample sets 1 and 2 were used to identify circadian RNA candidates (green), which were then validated in sample set 3 (blue). Relationships between CircaMiR levels and CircaMicrobes, or mRNA targets were explored (orange). The functional implications of CircaMiRs and CircaMicrobes were interrogated through annotation analyses and characterization in a cohort of children with disordered sleep (sample set 4; red). The relationship of oscillating RNA with patterns of daily activity (sleep, eating, and tooth brushing) were also investigated.

An overview of the sample sets and analyses is provided (Fig 1). Sample set 1 contained 24 saliva samples collected at 2 time-points (~9 AM, 9 PM) on 3 days from 4 participants. There were a total of 98 miRNAs in set 1 with a significant effect of collection time (FDR < 0.01) and no effect of day of collection (FDR > 0.05). Sample set 2 contained 48 samples collected at 4 time-points (~9 AM, 1:30 PM, 5:30 PM, 9 PM) on 4 days from 3 participants. There were a total of 123 miRNAs in set 2 that showed a significant effect of collection time and no effect of day. Levels of 61 miRNAs were similarly affected by time of collection in both sample sets and were defined as putative CircaMiRs (S1 Table).

**Fig 2:**
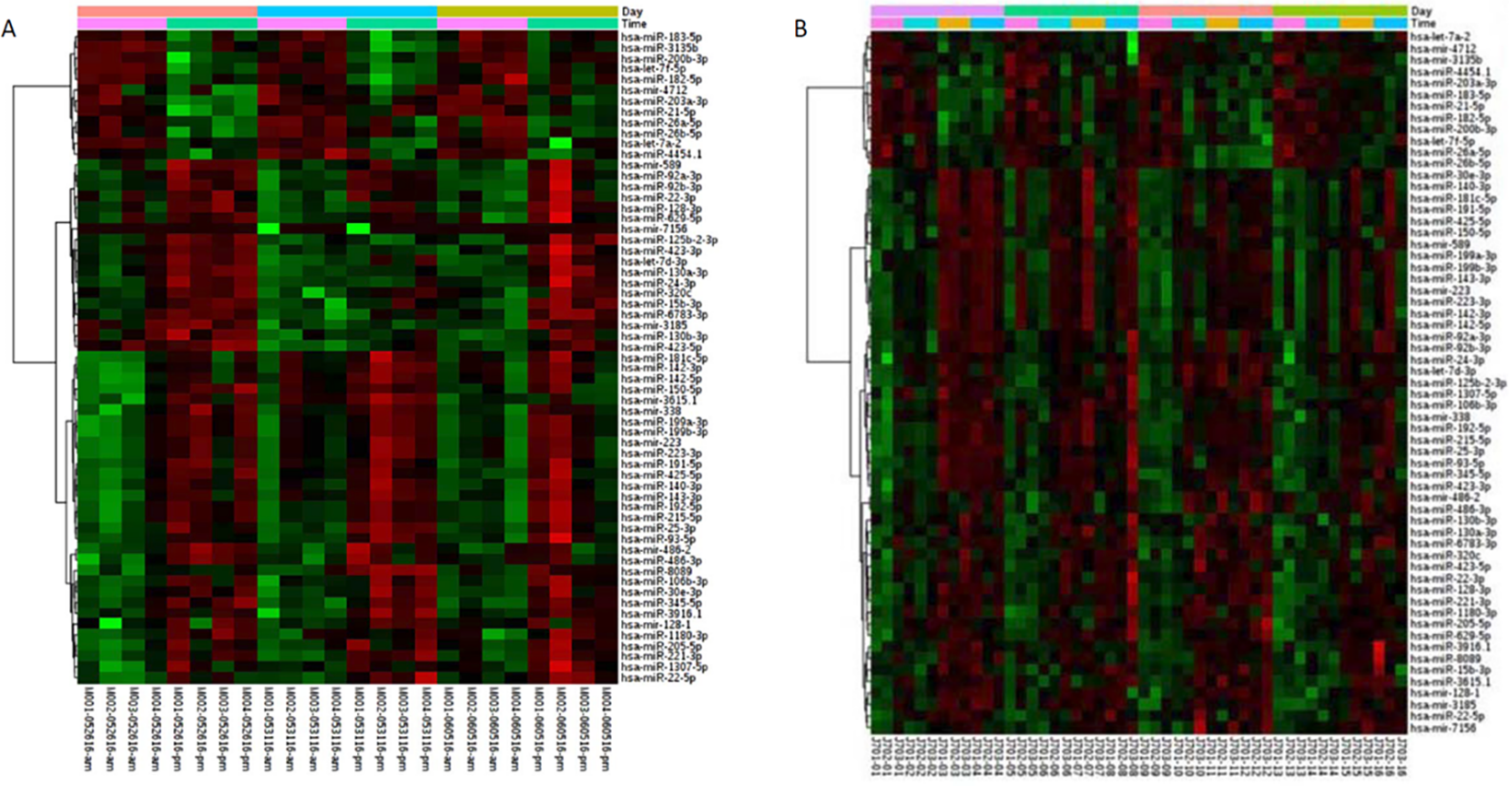
Salivary miRNA levels change across time. **(A)** Heat map clustering of expression data for the 61 miRNAs changed according to collection time in sample set 1. This set consisted of 24 samples from 4 subjects across 3 days of sampling (days 1, 3, 7) at a frequency of 2 times/day (9 am, 9 pm). **(B)** Heat map clustering of expression data for the 61 miRNAs changed according to collection time in sample set 2. This set consisted of 48 samples from 3 subjects obtained across 4 days of sampling (days 1, 5, 10, 15) at a frequency of 4 times/day (9 am, 1:30 pm, 5:30 pm, 9 pm).

Hierarchical (heat map) clustering using salivary concentrations of the 61 CircaMiRs was performed for sample set 1 (Fig 2A) and sample set 2 (Fig 2B). In both sample sets, the majority of CircaMiRs (n = 49; 80%) demonstrated lower levels in the morning and higher levels in the evening. Examination of the 61 miRNAs across four time points (sample set 2) revealed only a single oscillation (i.e. a single daily peak) between 9AM and 9PM. These daily oscillations were consistent across days of collection and across participants, as reflected by the lack of significant day effects in the two -way ANOVA (S1 Table).

**Fig 3:**
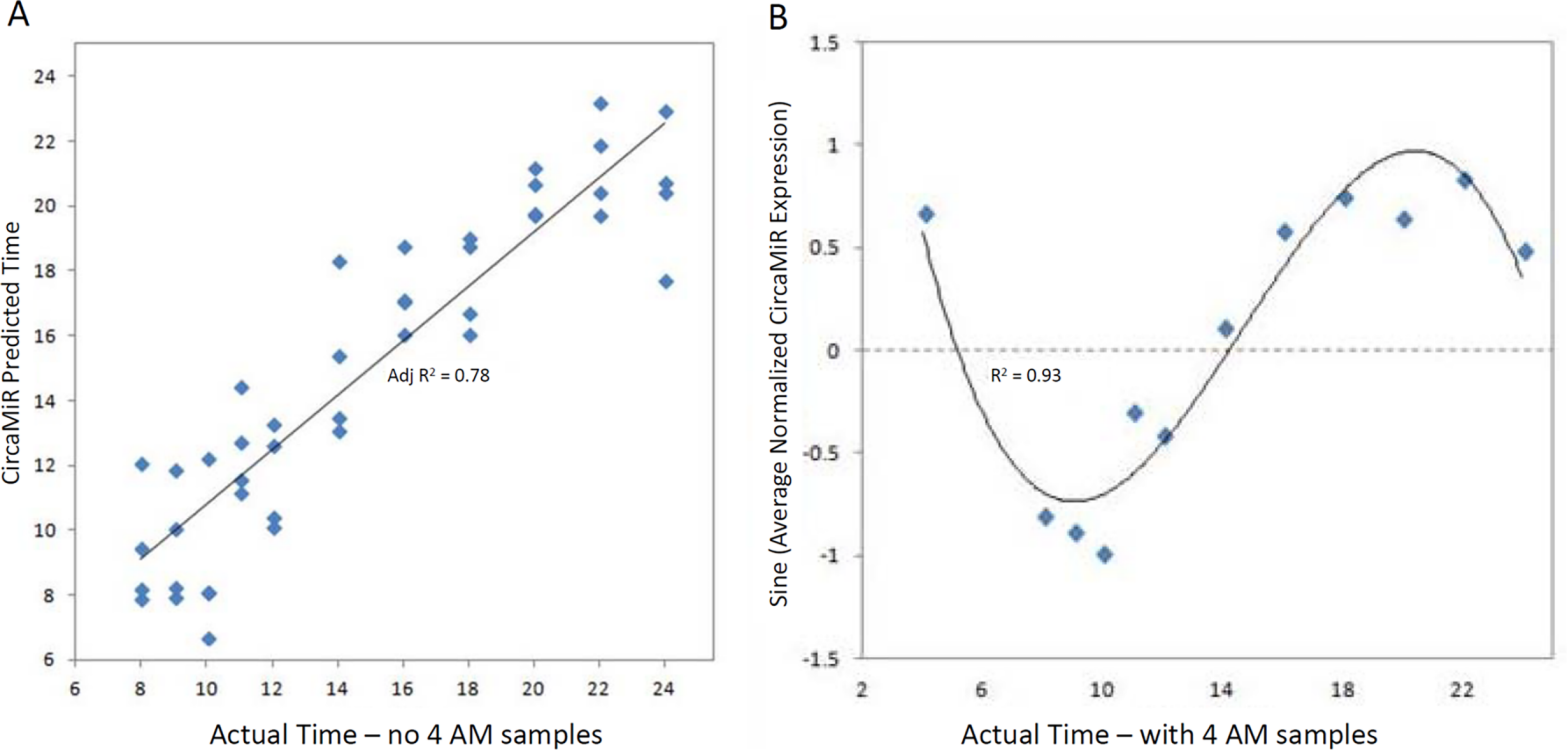
Accurate prediction of collection time from salivary miRNA levels. **(A)** 11 of the total 61 identified miRNA predictors and their accuracy of prediction for sample set 3. **(B)** Sine transformed values of the average expression of 1 of the 61 CircaMiRs (miR-199b-3p) for the subjects in sample set 3 (collected at various times across 2 days).

From the 61 CircaMiR candidates, 11 miRNAs were identified as robust multivariate predictors of collection time through a feature selection algorithm using a linear regression analysis. The regression model accurately predicted collection time in all 3 sample sets, with Multiple R values ranging from 0.805–0.956 and Adjusted R^2^ values ranging from 0.54–0.833 (Table 1). Notably, the multivariate model performed best when applied to samples collected during a wakeful state (9 AM–12 AM) and model performance significantly improved in sample set 3 when 4 AM samples were excluded (Adjusted R^2^ = 0.880 vs 0.794, Table 1, upper). This improvement was due to non-linear trends in the expression data during the overnight period (a circadian oscillation of high values back to low values and vice versa). In fact, the predictive utility of the linear regression model (R^2^ = 0.79; Fig 3A) was even found to be inferior to a non-linear regression model that used the *sine-transformed* average miRNA values for just one of the 61 CircaMiRs in the set 3 samples (R^2^ = 0.93; Fig 3B). Interestingly, further inspection of the alpha (intercept) and beta (slope) coefficient terms across the independent sample set regressions indicated a very high degree of internal consistency in these models (Table 2), with highly significant correlations present between all sets of model term comparisons except sample set 1 and sample set 3 with the 4 AM samples included.

**Table 1:** Salivary miRNA and microbial RNA model performance for predicting collection time.

	Multiple R	Adjusted R^2^	P-value	Mean Absolute Error (%)
***microRNAs***				
Sample set 1 (n = 24)	0.956	0.833	9.5E-05	9.2
Sample set 2 (n = 48)	0.805	0.540	1.8E-05	14.6
Sample set 3 (n = 48)	0.918	0.794	2.8E-11	12.7
Sample set 3 (no 4AM, n = 44)	0.954	0.880	1.1E-13	8.1
***microbial RNAs***				
Sample set 1 (n = 24)	0.927	0.732	0.0013	13.1
Sample set 2 (n = 48)	0.784	0.496	7.5E-05	15.0
Sample set 3 (n = 48)	0.770	0.468	1.8E-04	21.4
Sample set 3 (no 4AM, n = 44)	0.849	0.624	3.6E-06	15.1

**Table 2:** Correlations of salivary miRNA and microbial model terms across sample sets.

**miRNA model (11 beta coefficients + intercept)**				
	*Set 1*	*Set2*	*Set 3*	*Set 3 no 4 am*
*Set 1*		**0.7469**	0.5399	**0.7207**
*Set 2*	*0*.*0053*		**0.7060**	**0.8409**
*Set 3*	*0*.*0698*	*0*.*0103*		**0.9647**
*Set 3 no 4 am*	*0*.*0082*	*0*.*0006*	*<* .*0001*	
**Microbial RNA model (11 beta coefficients + intercept)**				
	*Set 1*	*Set2*	*Set 3*	*Set 3 no 4 am*
*Set 1*		**0.8929**	**0.8319**	**0.9066**
*Set 2*	*<* .*0001*		**0.8699**	**0.9542**
*Set 3*	*0*.*0008*	*0*.*0002*		**0.9630**
*Set 3 no 4 am*	*<* .*0001*	*<* .*0001*	*<* .*0001*	

### Salivary microbiome analysis

Sample set 1 contained a total of 75 microbial RNAs with a significant effect of collection time (FDR < 0.01) and no effect of day of collection (FDR>0.05). Sample set 2 contained a total of 32 microbial RNAs with a significant effect of collection time and no effect of day of collection. Eleven microbial RNAs with diurnal oscillations in sample sets 1 and 2 overlapped (Table 3). The 11 RNAs from these 11 distinct microbial species were defined as putative CircaMicrobes, and examined for their ability to predict collection time in sample set 3.

**Table 3:** List of 11 microbes most related to collection time.

		Sample set 1			Sample set 2		
Taxon ID	Taxon name	Day	Time	Interaction	Day	Time	Interaction
1510155	Falconid herpesvirus 1	0.7246	0.0003	0.1104	0.9999	0.0009	0.9982
553174	Prevotella melaninogenica ATCC 25845	0.8213	0.0011	0.1693	0.9999	0.0359	0.9982
862965	Haemophilus parainfluenzae T3T1	0.2276	0.0061	0.2426	0.9999	0.0045	0.9982
479436	Veillonella parvula DSM 2008	0.7246	0.0076	0.1069	0.9999	0.0001	0.9982
458233	Macrococcus caseolyticus JCSC5402	0.0830	0.0338	0.1302	0.9999	0.0381	0.9982
190304	Fusobacterium nucleatum subsp. nucleatum ATCC 25586	0.9782	0.0127	0.1069	0.9999	0.0350	0.9982
724	Haemophilus	0.5928	0.0127	0.2426	0.9999	0.0139	0.9982
469604	Fusobacterium nucleatum subsp. vincentii 3136A2	0.7246	0.0209	0.1069	0.9999	0.0187	0.9982
11855	Mason-Pfizer monkey virus	0.5439	0.0213	0.4616	0.9999	0.0046	0.9982
360107	Campylobacter hominis ATCC BAA-381	0.9713	0.0359	0.2413	0.9999	0.0084	0.9982
838	Prevotella	0.7246	0.0482	0.2844	0.9999	0.0037	0.9982

A multivariate linear regression model utilizing the 11 microbial RNAs was also able to accurately predict collection time in all 3 sample sets, with Multiple R values ranging from 0.770–0.927 and Adjusted R^2^ values ranging from 0.468–0.732 (Table 1). As with the miRNA model, a non-linear relationship between the time of collection and microbial RNA concentrations in sample set 3 reduced the overall accuracy of the microbial model across the full 24 hour time cycle compared to when the 4 am samples were removed from analysis (Adjusted R^2^ = 0.468 vs 0.624, Table 1), which yielded results comparable to those seen in sample sets 1 and 2. Likewise, inspection of the alpha (intercept) and beta (slope) coefficient terms across the independent sample set regressions again indicated a very high degree of internal consistency in these models with highly significant correlations present between all sets of model term comparisons (Table 2).

### Relationship between CircaMiRs and CircaMicrobes

**Fig 4:**
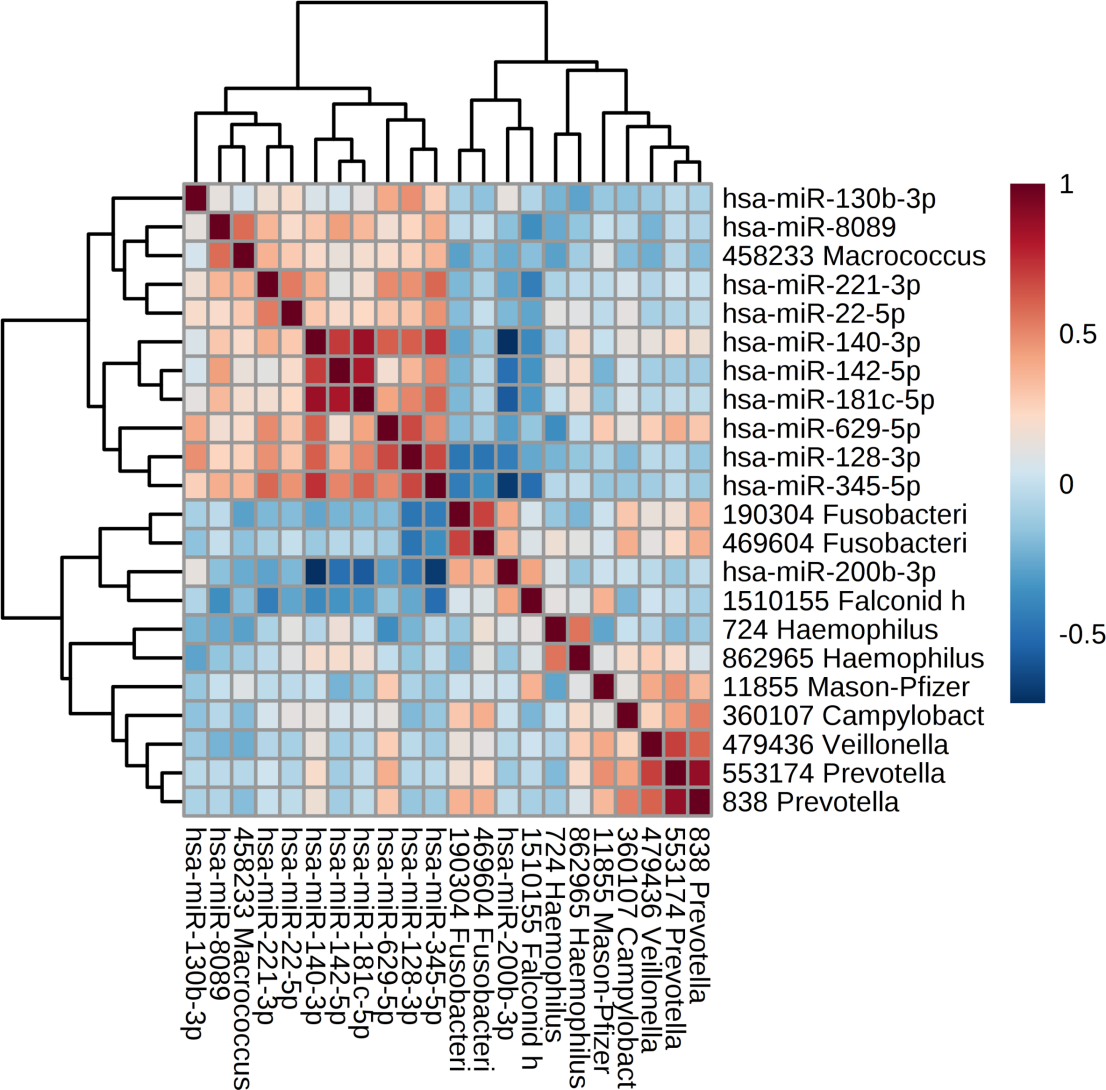
Quantitative relationships between CircaMiRs and CircaMicrobes. A Pearson’s correlation analysis was used to compare expression of 11 CircaMiRs and 11 CircaMicrobes. The 22 features are sorted by a complete clustering algorithm, and the hierarchical tree indicates similarity in expression pattern across samples. Blue indicates strong inverse relationships while red indicates strong direct relationships.

Relationships between levels of the 11 CircaMiRs and the 11 microbes with oscillating transcriptional activity were assessed across all 120 samples from sample sets 1, 2, and 3 using a Pearson’s correlation analysis. With the exception of one CircaMiR (miR-200b-3p) and one CircaMicrobe (Macrococcus caseolyticus), the CircaMiRs and CircaMicrobes were generally segregated by hierarchical clustering of expression patterns (Fig 4). However, 5/11 (45%) CircaMiRs and 4/11 (36%) CircaMicrobes demonstrated significant (|R|≥0.40, FDR<0.0001) associations. Three of these relationships involved direct associations (miR-8089/Micrococcus caseolyticus, miR-200b-3p/Fusobacterium nucleatum subsp. nucleatum, and miR-200b-3p/Falconid herpesvirus 1). There were five inverse associations between CircaMiRs and CircaMicrobes (miR-221-3p/Falconid herpesvirus 1, miR-128-3p/Fusobacterium nucleatum subsp. nucleatum, miR-128-3p/Fusobacterium nucleatum subsp. vincentii, miR-345-5p/Fusobacterium nucleatum subsp. nucleatum, miR-345-5p/Falconid herpesvirus 1).

### CircaMiR target genes

Functional analysis of the 11 CircaMiRs in DIANA miRPath revealed 1265 high confidence (p<0.05, Micro-T threshold ≥ 0.95) mRNA targets with enrichment for 22 KEGG pathways (Table 4). Notably, 11/22 KEGG pathway targets were involved in cell signaling. Interestingly, circadian rhythm was not among the KEGG pathways targeted by the 11 CircaMiRs according to this analysis. However, of the 30 human mRNAs in the circadian rhythm KEGG pathway (hsa04710), four (13%; Csnk1e, Rora, BHLHE40, and Prkaa2) were targeted by the 11 CircaMiRs. To more closely examine the potential relationship of CircaMiRs and circadian function, we expanded the analysis to the initial 61 CircaMiRs and used IPA software (which included additional circadian mRNA targets). The results revealed a significant overlap in Circadian Rhythm Signaling targets (13/34 mRNAs, 38%, p = 2.2e-38) based on moderate-to-high probability predicted interactions, or experimentally-observed interactions. A complete list of the 28 mRNA transcript isoforms encompassing the 13 mRNAs and their 37 CircaMiR interactors is provided (S2 Table).

**Table 4:** Physiologic pathways over-represented by mRNA targets of the 11 CircaMiRs.

KEGG pathway	p-value	#genes	#miRNAs
Rap1 signaling pathway	7.7E-05	30	9
Mucin type O-Glycan biosynthesis	1.5E-03	4	4
Ras signaling pathway	2.1E-03	30	8
Estrogen signaling pathway	2.1E-03	14	7
Lysine degradation	2.5E-03	6	6
ErbB signaling pathway	3.3E-03	17	7
PI3K-Akt signaling pathway	3.8E-03	38	9
Proteoglycans in cancer	4.7E-03	23	7
Neurotrophin signaling pathway	4.9E-03	19	7
Choline metabolism in cancer	5.2E-03	15	8
Renal cell carcinoma	1.2E-02	12	6
mTOR signaling pathway	1.5E-02	11	6
Prolactin signaling pathway	1.5E-02	11	7
MAPK signaling pathway	1.5E-02	29	8
FoxO signaling pathway	2.0E-02	17	7
Long-term potentiation	2.5E-02	11	7
Endocytosis	2.5E-02	21	8
Focal adhesion	3.6E-02	23	8
Oocyte meiosis	3.6E-02	14	7
Protein processing in endoplasmic reticulum	4.6E-02	18	5
Insulin signaling pathway	4.6E-02	17	5
Glutamatergic synapse	5.0E-02	13	5

The MiRpath target mapping tool also failed to detect enrichment of KEGG pathways involved in immune function or bacterial regulation among the 11 CircaMiR targets (or the 5 CircaMiRs with microbial associations in Fig 4). However, several of the CircaMiRs that mapped to circadian genes were found to target mRNAs that were clearly involved in immune function (S2 Table). Subsequent interrogation of the protein-protein interaction network for all 1127 unique mRNA targets of the 11 most robust CircaMiRs using STRING software, revealed 3794 edges (interactions) with a clustering coefficient of 0.32. This exceeds the number of protein-protein interactions expected by chance alone (p = 1.0E-16), and implies inter-relatedness of CircaMiR targets. Among the expected protein targets, 471 were involved in regulation of metabolic process (GO:0019222; FDR = 8.5E-23), 413 were involved in regulation of macromolecule metabolic process (GO:0060255; FDR = 4.9E-22), and 425 were involved in regulation of cellular metabolic process (GO:0031323; FDR = 8.9E-22; S3 Table).

### Transcript overlaps

Of the 1265 mRNAs targeted by the 11 CircaMiRs with high confidence (micro-T-cds score ≥ 0.950), 38 were reliably detected in saliva (counts ≥ 10 in 10% of samples) with small RNA sequencing at 50 base pairs. Among these 38 mRNAs, the salivary levels of 8 (21%) were significantly associated (FDR<0.05) with their CircaMiR counter-parts (Table 5). Two mRNAs were positively associated with miR-130b-3p (ATXN1, FOSL2), three were positively associated with miR-142-5p (GRIN2B, MSL2, NAMPT), one was negatively associated with 181c-5p (WASL), and two were positively associated with miR-200b-3p (YOD1, YWHAG). The strongest relationship was observed between miR-142-5p and GRIN2B (R = 0.53, FDR = 8.71E-09, Target score = 0.984), a member of the Circadian Rhythm Signaling pathway in IPA.

**Table 5:** Transcripts targeted by CircaMiRs with associated expression levels across time.

MicroRNA	Gene	R	T-stat	p-value	FDR	Micro-CDS Target Score
miR-130b-3p	ATXN1	0.37395	4.3799	2.59E-05	0.000129	0.963
miR-130b-3p	FOSL2	0.49302	6.1557	1.06E-08	1.23E-07	0.969
miR-142-5p	GRIN2B	0.53012	6.7914	4.76E-10	8.71E-09	0.984
miR-142-5p	MSL2	0.42696	5.129	1.16E-06	6.49E-06	0.981
miR-142-5p	NAMPT	0.51006	6.4417	2.67E-09	3.5E-08	0.969
miR-181c-5p	WASL	-0.2945	-3.3476	0.001094	0.002056	0.966
miR-200b-3p	YOD1	0.23098	2.5788	0.011142	0.031478	0.973
miR-200b-3p	YWHAG	0.29093	3.3032	0.001266	0.005204	0.985

### Metabolic targets of the oral microbiome

**Fig 5:**
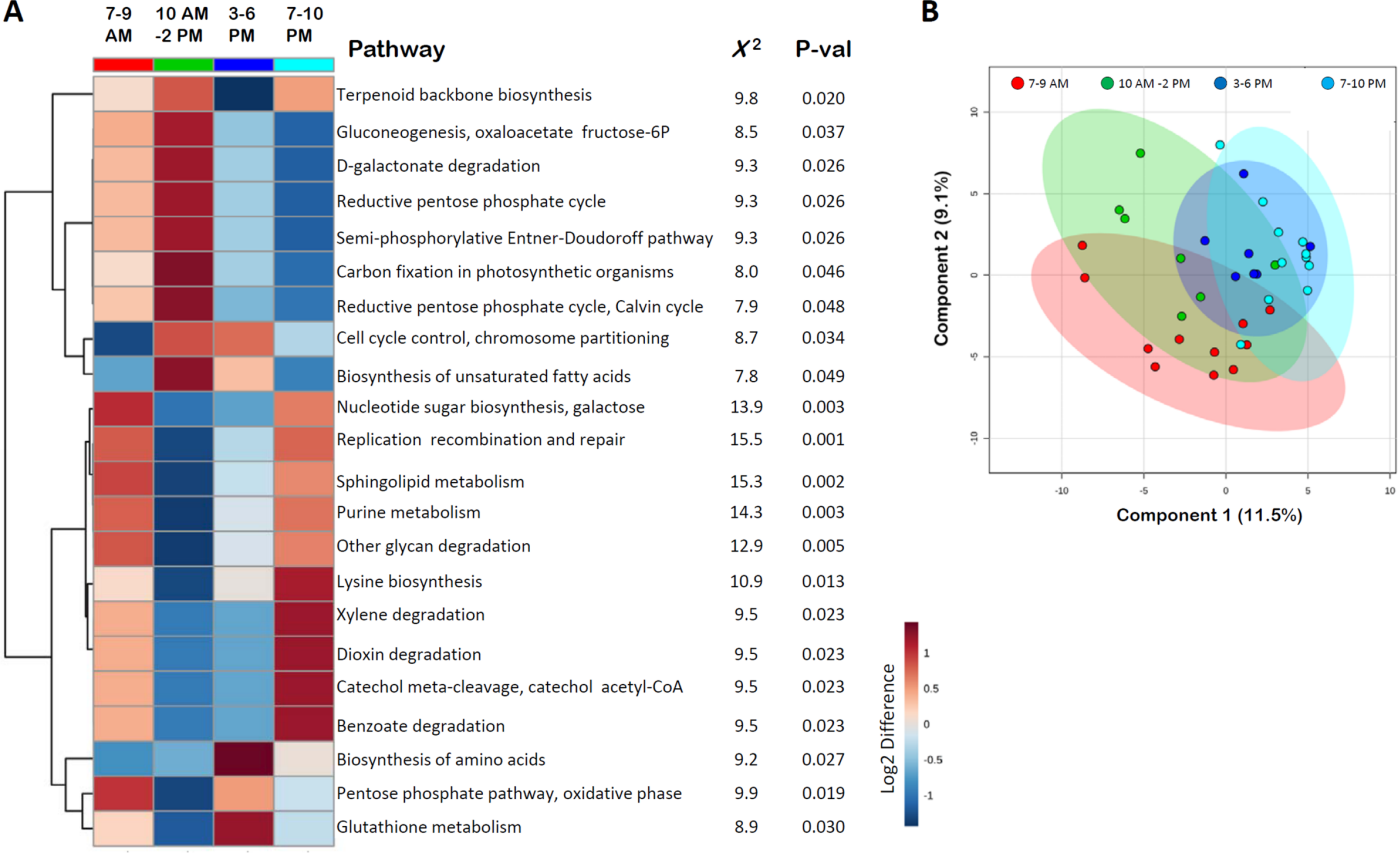
Changes in functional microbiome expression across time. **(A)** The hierarchical heat map displays average abundance values for microbial RNAs representing 22 KEGG/COG metabolic pathways, that displayed nominal differences (p<0.05) in expression across 4 time periods (7–9 AM, 10 AM-2 PM, 3–6 PM, 7–10 PM). The dendrogram (y-axis) represents inter-relatedness of KEGG/COG pathway activity measured by Pearson distance metric across the 120 samples. Red denotes relative increased abundance of KEGG/COG transcripts, while blue denotes relative decrease in related transcripts. Chi-square and raw p-values (Kruskal-Wallis ANOVA) are displayed for each of the 22 pathways. **(B)** A partial least squares discriminant analysis utilizing mean abundance levels for all 202 KEGG/COG metabolic pathways with microbial RNA mappings is displayed for the four collection time periods. Note that global metabolic activity in these 202 pathways achieves partial separation of the four time periods, while accounting for 20.6% of the variance in the dataset.

RNA expression patterns of oral microbes from the 9 participants in sample sets 1, 2, and 3 were examined for evidence of diurnal variations in metabolic and functional clusters across four time periods: 7–9 AM, 10 AM–2 PM, 3–6 PM, and 7–10 PM. Among the 202 functional clusters targeted by microbial RNAs, 22 pathways demonstrated nominal (p<0.05) differences in representation across the four time periods (Fig 5A). Four of these functional pathways (nucleotide sugar biosynthesis, galactose; replication recombination and repair; sphingolipid metabolism; and purine metabolism) survived multiple testing corrections (FDR≤0.15). Among the 22 functional pathways with nominal changes, a cluster of seven pathways was up-regulated mid-day (10 AM–2 PM), while 10 pathways demonstrated diurnal peaks in the morning (7-9AM) and evening (7–10 PM). Visualization of functional pathway expression differences in a partial least squared discriminant analysis resulted in partial separation of the four time periods, while accounting for 20.6% of the variance in COG/KEGG data in two dimensions (Fig 5B).

### Measuring CircaMiR levels in children with disordered sleep

**Fig 6:**
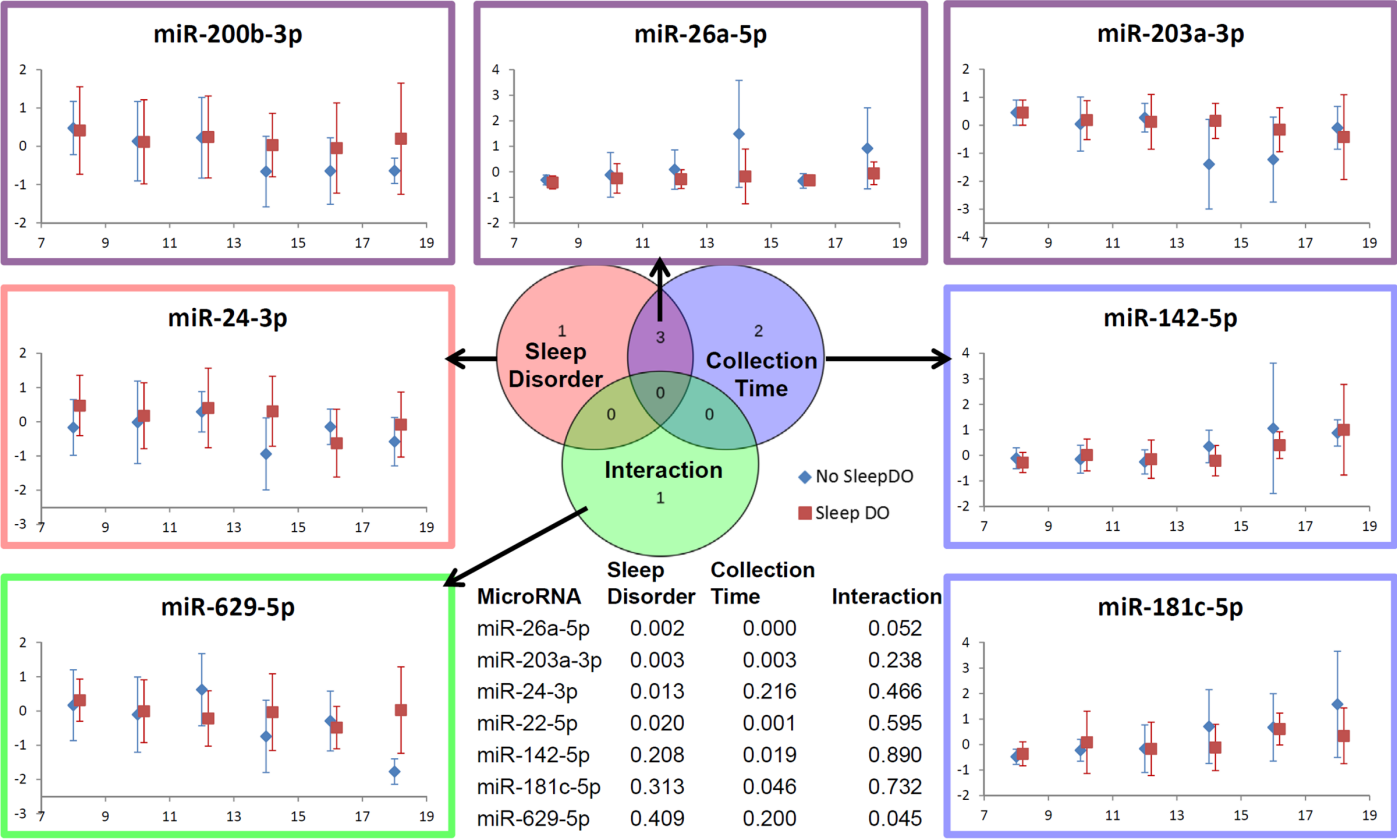
Relationships between CircaMiRs, collection time, and sleep disorder in children with autism spectrum disorder. A two-way ANOVA was performed to assess the relationship between CircaMiR expression level, collection time, and the presence/absence of disordered sleep in a cohort of 140 children with autism spectrum disorder The Venn diagram (center) shows that 7/14 (50%) of theses CircaMiRs displayed significant relationships with collection time, disordered sleep, or a time-sleep interaction. Mean expression level at 6 time points (8-9AM, 10-11AM, 12-1PM, 2-3PM, 4-5PM, and 6-8PM) is displayed for participants with (red), or without (blue) disordered sleep for each of the 7 CircaMiRs of interest. Two-way ANOVA p-values are listed for each CircaMiR in the embedded table (center, bottom).

Differences in salivary miRNA expression between autistic children with (n = 77) and without (n = 63) disordered sleep was assessed with Mann Whitney U-test. Among the 61 CircaMiRs three demonstrated differences (FDR<0.05) between the two groups (miR-26a-5p, miR-24-3p, miR-203a-3p; S4 Table). Because this approach could not account for phase shifts in diurnal miRNA expression, salivary miRNA levels in the ASD cohort were also assessed with a 2-way ANOVA accounting for sleep disorder diagnosis and saliva collection time. This ANOVA analysis included the 11 robust CircaMiRs and the three miRNAs identified on Mann-Whitney testing. Among these 14 miRNAs, 4 demonstrated a significant interaction (p<0.05) with sleep disorder diagnosis (miR-24-3p, miR-200b-3p, miR-203a-3p, miR-26a-5p), 5 demonstrated a significant interaction with collection time (miR-142-5p, miR-181c-5p, miR-200b-3p, miR-203a-3p, miR-26a-5p), and 3 were affected by both factors (Fig 6). We also detected a significant interaction between collection time and sleep disorder diagnosis for one CircaMiR (miR-629-5p).

**Fig 7:**
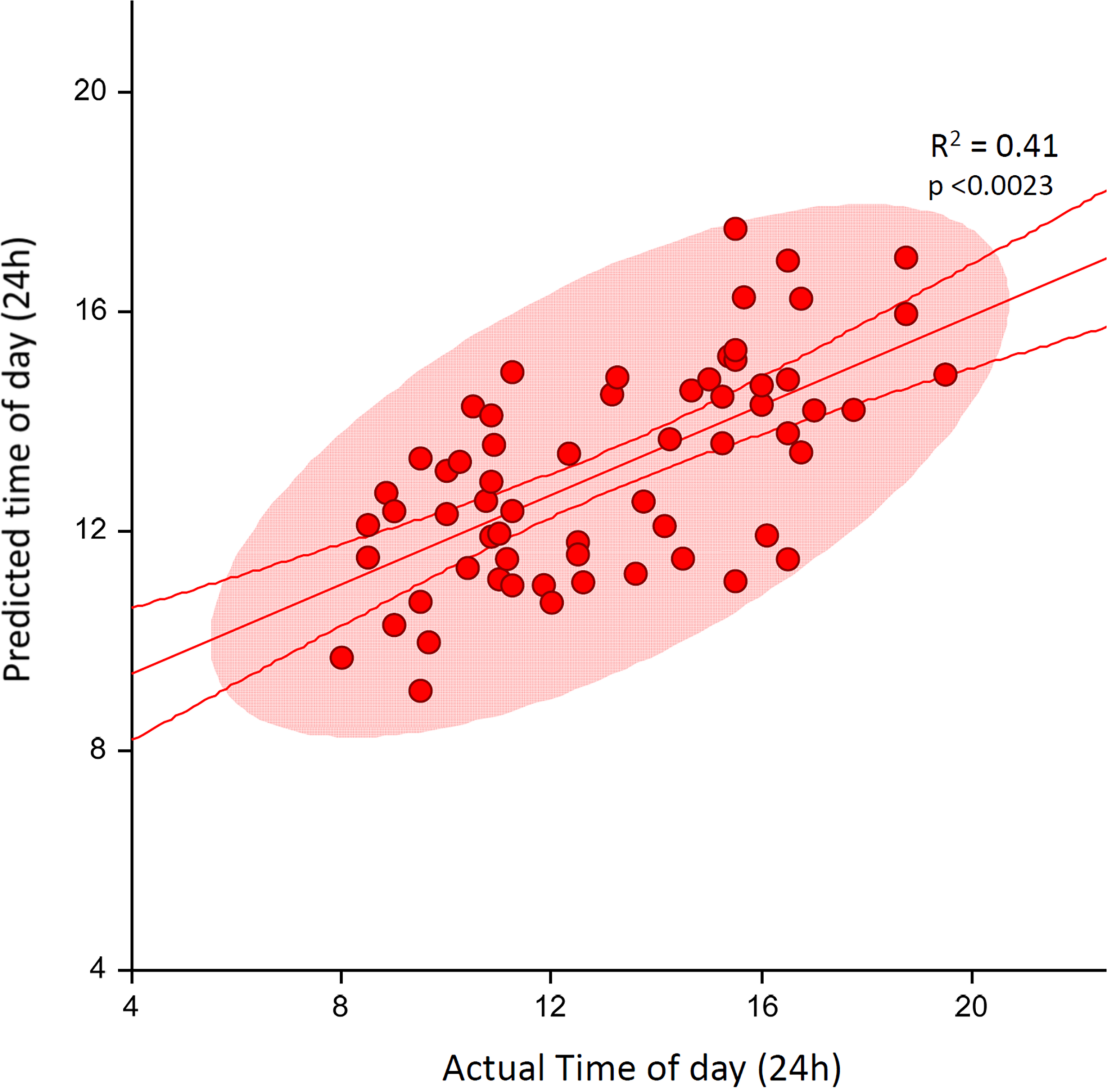
Multivariate regression with CircaMiRs predicts collection time in sample of children with autism spectrum disorder. The graph plots the relationship between the predicted time and actual time in hours for a subset of 63 autistic children who had normal sleep patterns using 11 CircaMiRs. The lines above and below the regression line indicate the 95^th^ confidence interval of the fitted regression. The colored ellipse represents the 95^th^ confidence interval of the actual data points. Note that there was an absence of a significant relationship in autistic children with a sleep disorder (not shown).

Based on the ability of the 11 CircaMiRs to predict time of collection in 11 typically developing, healthy children (and adults) in sample sets 1, 2 and 3, we also used a multivariate regression model examining their ability to predict time of collection in the 63 children with ASD and a normal sleep pattern, and the 77 children with ASD and comorbid disordered sleep. As we had seen in sample sets 1, 2 and 3, these 11 CircaMiRs yielded a significant regression (R^2^ = 0.41, F_1,11_ = 3.19, p <0.0023) that accurately predicted the time of collection with a mean absolute error of 15.25% (Fig 7). Inspection of the multivariate regression coefficients and T scores indicated that no individual miRNA was significant in the presence of the others, although three showed strong trends (miR-629-5p, miR-22-5p, and miR-128-3p) (Table 6). In contrast to the significant regression findings for the ASD children without sleep disorder (n = 63), the regression results for the ASD children with sleep disorders (n = 77) using the 11 CircaMiRs did not yield a significant result (R^2^ = 0.20, F_1,11_ = 1.46, p >0.167).

**Table 6:** Prediction of collection time in ASD children with normal sleep patterns (n = 63).

Variable	T-stat	P	Power
hsa-miR-128-3p	1.824	0.074	0.432
hsa-miR-130b-3p	0.055	0.956	0.050
hsa-miR-140-3p	-0.250	0.803	0.057
hsa-miR-142-5p	0.794	0.431	0.122
hsa-miR-181c-5p	0.489	0.627	0.077
hsa-miR-200b-3p	-0.892	0.377	0.141
hsa-miR-22-5p	-1.860	0.069	0.446
hsa-miR-221-3p	-0.899	0.373	0.143
hsa-miR-345-5p	-0.110	0.913	0.051
hsa-miR-629-5p	-1.961	0.055	0.486
hsa-miR-8089	0.983	0.331	0.161

### Relationships of CircaMiRs and CircaMicrobes with daily activities

Pearson’s correlation analysis was used to explore relationships between oscillating salivary RNAs and three daily routines (sleep, tooth brushing, and eating) in sample set 3. Levels of 3 CircaMiRs and 5 CircaMicrobes were significantly (FDR<0.05) associated with time since last sleep (measured in hours; Table 7). Levels of all five CircaMicrobes were *inversely* associated with time since sleep, while 2/3 CircaMiRs were *positively* correlated with time since last sleep. There were 4 CiraMiRs and 5 CircaMicrobes associated with time since last tooth brushing. Levels of all five CircaMicrobes were *inversely* associated with time since last tooth brushing, while 3/4 CircaMiRs were *positively* associated with time since last tooth brushing. MiR-200b-3p was the single CircaMiR inversely associated with both sleep and tooth brushing. Notably, expression patterns of miR-200b-3p were also hierarchically clustered with CircaMicrobe expression (Fig 4). Two Prevotella and two Fusibacterium CircaMicrobes were associated with both sleep and tooth brushing. There was only one CircaMicrobe positively associated with time since last meal (and 0 CircaMiRs).

**Table 7:** Relationship between oscillating salivary RNA levels and timing of daily activities.

RNA Component	Hours awake since last sleep			Hours since last toothbrush			Hours since last meal		
	R	FDR	T-stat	R	FDR	T-stat	R	FDR	T-stat
***Microbial RNA***									
Falconid herpesvirus 1	-0.48	0.00	-3.75	-0.18	0.36	-1.23	0.09	0.75	0.63
Prevotella melaninogenica ATCC 25845	-0.39	0.03	-2.88	-0.50	0.00	-3.92	0.23	0.33	1.58
Haemophilus parainfluenzae T3T1	0.18	0.40	1.21	0.24	0.19	1.69	0.01	0.97	0.07
Veillonella parvula DSM 2008	-0.36	0.05	-2.60	-0.15	0.44	-1.06	0.36	0.11	2.59
Macrococcus caseolyticus JCSC5402	0.28	0.14	1.96	0.30	0.09	2.12	0.02	0.94	0.16
Fusobacterium nucleatum subsp. nucleatum 25586	-0.57	0.00	-4.76	-0.61	0.00	-5.24	0.24	0.30	1.69
Haemophilus	0.17	0.41	1.19	0.32	0.07	2.30	-0.04	0.90	-0.27
Fusobacterium nucleatum subsp. vincentii	-0.46	0.01	-3.51	-0.50	0.00	-3.88	0.29	0.21	2.07
Mason-Pfizer monkey virus	-0.35	0.05	-2.56	-0.18	0.36	-1.24	0.38	0.09	2.77
Campylobacter hominis ATCC	-0.24	0.22	-1.67	-0.47	0.00	-3.58	0.44	0.04	3.34
Prevotella	-0.47	0.01	-3.58	-0.60	0.00	-5.03	0.30	0.19	2.13
***Human microRNA***									
miR-142-5p	0.37	0.04	2.71	0.42	0.01	3.13	-0.16	0.53	-1.09
miR-130b-3p	0.12	0.60	0.81	-0.04	0.87	-0.26	-0.22	0.34	-1.55
miR-629-5p	-0.04	0.88	-0.26	-0.29	0.11	-2.04	0.05	0.88	0.32
miR-140-3p	0.35	0.05	2.55	0.23	0.23	1.57	-0.35	0.12	-2.49
miR-128-3p	0.35	0.05	2.54	0.30	0.09	2.11	-0.35	0.12	-2.50
miR-181c-5p	0.25	0.19	1.76	0.16	0.42	1.10	-0.37	0.09	-2.70
miR-345-5p	0.70	0.00	6.70	0.58	0.00	4.83	-0.38	0.09	-2.78
miR-22-5p	0.35	0.05	2.50	0.35	0.04	2.52	0.01	0.98	0.04
miR-8089	-0.01	0.96	-0.09	0.08	0.70	0.55	0.11	0.68	0.77
miR-221-3p	0.21	0.30	1.45	0.07	0.75	0.46	0.03	0.92	0.23
miR-200b-5p	-0.44	0.01	-3.31	-0.43	0.01	-3.26	0.21	0.39	1.44

## Discussion

In the present study, 61 total human miRNAs (CircaMiRs) and 11 total microbes (CircaMicrobes) displayed consistent diurnal oscillations in saliva samples obtained from 9 different children and adults collected across multiple days and times. From these, 11 miRNAs and 11 microbes were capable of accurately and reliably predicting time of sample collection. Diurnal levels of five CircaMiRs and four CircaMicrobes were strongly associated with one another. Functional analyses of the circadian RNA components displayed enrichment for numerous signaling mechanisms, particularly metabolic pathways. However, CircaMiR and CircaMicrobe levels were more strongly associated with sleep routines than with eating routines. This may explain, partly, why levels of four CircaMiRs were “altered” in autistic children with disordered sleep, relative to autistic peers without a sleep disorder and why a portion of these CircaMiRs target circadian mRNAs.

Six of the oscillating miRNAs identified in this study (miR-15b-3p, miR-24-3p, miR-106b-3p, miR-140-3p, miR-150-5p, miR-203a-3p) were among the 26 plasma miRNAs previously found to have diurnal variations in peripheral blood samples from healthy individuals [24]. These overlapping miRNAs from two distinct biofluids may represent either primary regulatory elements or primary readouts of circadian rhythmicity. This premise is supported by the fact that levels of both miR-24-3p and miR-203a-3p are disrupted in the cohort of autistic children with disordered sleep patterns. In addition, we found suggestive evidence that miR-203a-3p was associated with sleep initiation difficulties (R = 0.20; p = 0.034). Another CircaMiR, miR-142-5p, targets the clock gene RORA. Notably, miR-142-5p also displays correlated diurnal expression with its mRNA targets NAMPT (whose gene product modulates circadian clock function by releasing the CLOCK/ARNTL/BMAL heterodimer [25]) and GRIN2B (whose gene product encodes the NR2B subunit of the NDMA receptor essential to MAPK signaling in the suprachiasmatic nucleus and CaMK II signaling in the hippocampus [26]). Notably, a well-described developmental switch from NR2A to NR2B subunit expression is considered a hallmark of synaptic maturation that promotes memory formation, and elevation in miR-142-5p (which would suppress NR2B expression) is associated with amyloid beta pathology in postmortem brain samples of subjects with Alzheimer’s disease (AD) [27]. The importance of this finding is highlighted by the fact that AD is associated with significant circadian pathology (e.g. “sundowning”) and that miR-142-5p restores normal synapse formation and maturation (as measured by PSD95 expression) in differentiated neural cultures [28]. Such a mechanism might even contribute to the recently described circadian oscillation in synaptic spine number that has been described across different species, especially dendritic spines on inhibitory neurons in multiple brain regions [29–31].

Circadian miRNAs found in both plasma and saliva may also direct diurnal physiologic processes common to both peripheral biofluids. Indeed, mapping of KEGG pathway targets for the six overlapping miRNAs reveals enrichment for broad signaling mechanisms such as Wnt Signaling, Rap1 signaling, and Endocrine factor-regulated calcium reabsorption. This is consistent with functional analysis of the 11 CircaMiRs, which also display enrichment for Rap1 and other broad signaling processes (Ras, ErbB, PI3K-Akt, mTOR, MAPK). Salivary CircaMiRs also demonstrate target enrichment for endocrine factors (estrogen and prolactin signaling), which regulate peripheral physiologic processes in a circadian manner [32].

Unlike oscillating miRNAs in plasma, the CircaMiRs and CircaMicrobes in saliva appear uniquely geared toward metabolic functions. CircaMiR targets display enrichment for lysine degradation, choline metabolism, and insulin signaling (Table 3). The protein products of these mRNA targets also exhibit enhanced biologic interaction in metabolism at the cellular and macromolecule levels (S3 Table). Specifically, interactions between miR-130b-3p/ATXN2, and miR-142-5p/NAMPT (Table 4) may play important roles in regulation of host metabolism, given that loss of function mutations in both ATXN2 and NAMPT are associated with obesity and diabetes mellitus [33, 34].

Oscillating RNA expression within the oral microbiome also shows relationships with diurnal metabolism. Microbial RNAs appear to target KEGG and COG pathways in a diurnal manner, by up-regulating RNAs involved in terpenoid biosynthesis, gluconeogenesis, pentose phosphate pathways, and carbon fixation during the morning and afternoon time periods. In comparison, pathways related to cell replication, nucleotide biosynthesis, and purine metabolism demonstrate both morning and evening peaks. Thus, as a whole, the oral microbiome may have evolved energy utilization patterns that capitalize on the timing of host meals to extract biosynthetic materials and allow for night time replication. Interestingly, however, levels of the 11 CircaMicrobes do not appear to correlate with time since last meal. Thus, these 11 individual entities may serve a more commensal function whose metabolic activities aid host circadian rhythms. Indirect evidence for this may be found in the circadian rhythm of terpenoid biosynthesis (Fig 5A), a diverse class of hydrocarbons present in plant-based cannabinoids, or anti-inflammatory curcuminoids that play an essential role in steroid production [35]. Given the well-established rhythmicity of steroid production, this is one mechanism by which the microbiome may contribute to host circadian biology [36].

Further evidence for a synergistic relationship between CircaMicrobes and human hosts is found in the strong associations among CircaMiR and CircaMicrobe expression (Fig 4). It is somewhat surprising that CircaMiRs have few immune, or antimicrobial targets. However, this may be because the circadian components of the oral microbiome serve a commensal function. The majority of CircaMicrobes are not known to play pathogenic roles in human hosts. Of the 11 CircaMicrobes, only three are distinct human pathogens (Haemophilus parainfluenza T3T1, Haemophilus, and Campylobacter hominis ATTC BAA-381) and none of these three are associated with CircaMiR levels. Instead CircaMiRs may interact with the oral microbiome to coordinate metabolic patterns, or production of essential amino acids. Perhaps metabolic activity by the oral microbiome leads to changes in host miRNAs that regulate downstream physiologic pathways.

To our knowledge this is only the second study to report consistent circadian rhythmicity of peripheral miRNA expression in humans, and the first to do so in saliva. The importance of this finding is underscored by the vast number of publications seeking to use peripheral miRNA expression as a biological marker of human disease [37], a venture that could be greatly confounded by failure to control for time of collection. For example, among seven studies describing peripheral miRNA expression (saliva, serum, lymphoblasts [38]) in patients with ASD, none have reported, or controlled for time of RNA collection. These studies have reported a combined 139 ASD-related miRNAs. Notably, 10 of these (7%) overlap with the 61 CircaMiRs identified herein, which could represent confounded results. Future studies may be able to utilize CircaMiR levels to control for circadian variation in miRNA expression or accurately identify time of collection among biofluid samples.

The current study also adds to the growing body of literature that suggests miRNAs may serve as a communication mechanism between the gut microbiome and human hosts [39]. Specifically, these results show how miRNA-microbiome cross-talk may occur in a circadian manner. Given the diurnal rhythmicity of human metabolism, this finding has implications in human health and disease. For instance, daily fluctuations in host-microbiome interaction may inform risk for obesity, or insulin resistance (an enriched KEGG target of the 11 CircaMiRs). Alternatively, disruptions in miRNA-microbiome networks may unsettle the gut-brain-axis, a concept implicated in diseases such as Parkinson’s [40] and ASD [41] (both of which are associated with disordered sleep).

There are several notable limitations to this study that must be considered in interpreting these findings. Detailed daily activity logs were available only for the participants in sample set 3. The remaining participants reported no medical co-morbidities (including disordered sleep), though timing of sleep initiation and cessation were not recorded. Such information, when recorded alongside physiologic measurements such as sleep architecture, or melatonin flux could be extremely informative when interpreting RNA results in future studies. Nonetheless, the RNA expression patterns from participants in sample sets 1 and 2 were sufficient to accurately predict collection time in a third independent sample set with documented sleep wake cycles.

Notably, predictive performance in sample set 3 was somewhat impaired for the subset of samples obtained at 4 AM. This may have resulted because the sinusoidal model created from samples collected between 8 AM and 8 PM could not fully account for the overnight rhythmicity that occurs in a sleep state. There may also be microbial variability introduced by differences in participant breathing patterns (e.g. open-mouthed versus nasal breathing) or fasting during sleep. Certainly, a more controlled study which tightly dictated wake time, sleep initiation time, diet, dental hygiene, and other factors could account for time of collection with greater precision. However, the current results demonstrate that even in the face of typical variability among daily routines, these 11 miRNAs and 11 microbial RNAs are remarkably accurate predictors of time of saliva collection in four different cohorts of human subjects.

The accuracy of these results may even be underestimated given the broad age range (3–55) of participants in sample sets 1, 2, and 3. The CircaMiR and CircaMicrobe candidates were generated from 2 cohorts of children and validated in a cohort of teens and adults. This is despite the fact that teens are known to have altered circadian rhythm compared with pre-teen peers and adults[42]. Circadian RNAs from sample sets 1–3 also demonstrated significant relationships with collection time in a large cohort of children with ASD. Thus, the age and developmental diversity of these sample sets may be viewed as a confounding variable, but it likely enhances the veracity of these results.

Finally, it should be noted that the RNAseq approach used to identify oral microbes and estimate transcriptional activity of individual taxons differs from the typical 16S approach used to measure microbial abundance. Thus, these results should not be interpreted as diurnal fluctuations in the quantity of the oral microbiome, but rather as circadian variation in salivary microbial activity. RNAseq and 16S measures are complementary (though not equivocal) and could potentially add to the interpretive value of this approach in future studies. Such studies might also utilize animal models to explore the cellular origins of salivary CircaMiRs, and investigate the mechanisms regulating CircaMiR production, transport, and degradation. Manipulating the gut microbiome in this setting may also provide insights into microbial-miRNA communication.

Parallel circadian oscillation in host and microbial RNA represents an important consideration for studies analyzing epi-transcriptomic or metagenomic mechanisms in human health and disease. Circadian rhythm disturbances are a common problem in disorders of the central nervous system (e.g. Parkinson's, Alzheimer’s, autism, depression, concussion [43]). Hence, studies of peripheral miRNA expression in these conditions might consider how diurnal miRNA expression patterns are shifted, rather than simply focusing on average miRNA levels at a single collection point in comparison with a control cohort. Monitoring levels of these factors in biofluids like saliva could have diagnostic potential in diseases with altered circadian rhythm and may one day provide a basis for targeted miRNA therapy of circadian disruptions.

## Methods

### Subject assessment

This study was approved by the Institutional Review Board for the Protection of Human Subjects (IRB) at SUNY Upstate Medical University. Informed written consent was obtained for nine healthy human volunteers, and verbal assent was provided by all participating children.

### Study design

A prospective cohort design employing high throughput RNA sequencing was used to examine salivary RNAs (human and microbial) for daily oscillations in concentration (Fig 1). Nine healthy participants (3–55 years of age) were divided into three groups, and provided multiple saliva samples across a unique multi-day timeline (described below). Overlapping circadian RNA candidates from the first two independent sample sets were validated in a third sample set. Human miRNAs and microbial RNAs with confirmed diurnal variation were examined for associations in expression levels. Relationships between oscillating miRNAs and coding mRNA targets were also explored. Finally, the circadian RNA components were interrogated for functional relevance to human health and disease with the following three steps: 1) mRNA target networks for human miRNAs were identified in DIANA miRPath and Ingenuity Pathway Analyst (IPA, Qiagen), while metabolic pathways targeted by microbial RNAs were defined with MicrobiomAnalyst; 2) oscillating RNAs were retrospectively interrogated in a cohort of 140 children with autism spectrum disorder (ASD) with comorbid (n = 77), or absent (n = 63) sleep disturbance; and 3) the relationship of diurnal salivary RNAs with daily activities (tooth brushing, sleep, and eating) was assessed through Pearson correlation testing.

This study examined human miRNA and microbial RNA in saliva, because this biofluid provides on-demand access to repeated sampling of the GI tract at its sole point of entry, and represents a major site of host-environment interaction. Furthermore, studies of salivary miRNA in human patients have previously shown connections with brain-related dysfunction and potential relationships with time of collection [44, 45].

### Participants

Participants included nine healthy volunteers, taking no daily medications, with no history of hospitalization, surgery, or sleep disorder. None of the participants had active dental caries. The nine participants were 3–55 years of age, 55% male, and 100% Caucasian. Participants provided saliva samples at various times of day on repeated days in four different sets of samples:

Sample Set 1: Morning and evening samples (n = 24) collected at approximately 9 AM and 9 PM on days 1, 3, and 7 for 4 children (two male, two female; average age 7.5 yrs);

Sample Set 2: Early morning, early afternoon, late afternoon, and early evening samples (n = 48) collected at approximately 9 AM, 1:30 PM, 5:30 PM, and 9 PM on days 1, 5, 10 and 15 for three female children (average age 5.1 yrs), of whom two were part of Sample Set 1;

Sample Set 3: 12 samples collected at various times (ranging from 4 AM to midnight) on days 1 and 2 on two male children (average age 16.0 yrs) and their male and female parents (average age 51.5 yrs). Notably, detailed data regarding time of sleep, meals, and tooth brushing was collected for participants in Sample set 3.

Sample Set 4: Functional analysis of circadian RNAs was performed through retrospective analysis of data from an additional cohort of 140 children with ASD and comorbid sleep disturbance (n = 77), or normal sleep (n = 63). Salivary RNA was collected from these 140 children (2–6 years of age) at a single time-point, between 8 AM and 8 PM in a non-fasting state. ASD was confirmed through physician diagnosis, using the Diagnostic and Statistics Manual of the American Psychiatric Association, 5^th^ Edition (DSM-5) criteria. Disordered sleep was identified through parent survey and chart review by research staff. Participants with disordered sleep had either: 1) parent reported difficulty with sleep initiation or sleep maintenance; 2) ICD-10 diagnosis of disordered sleep (G47 or F51); or 3) a prescription for melatonin, clonidine, or mirtazapine with indication as a sleep aid. There was no difference in mean collection time between ASD subjects with (12:30PM ± 2:48) and without (1:00PM ± 3:00) disordered sleep (p = 0.34). The sleep disorder group was 18% female (14/77) and had a mean age of 56 (±16) months. The non-sleep disorder group was 14% female (9/63) and had a mean age of 56 (± 13) months.

### Saliva collection and processing

Before collecting saliva samples, each subject rinsed their mouth with tap water. Approximately 1 mL of saliva was obtained through swab collection using an Oragene RNA collection kit (DNA Genotek; Ottawa, Canada). Samples were stored at room temperature until processing. A Trizol method was used to purify the salivary RNA and a second round of purification was followed using an RNEasy mini column (Qiagen). Yield and quality of the RNA samples was assessed with the Agilent Bioanalyzer. This was done prior to library construction in accordance to the Illumina TruSeq Small RNA Sample Prep protocol (Illumina; San Diego, California). Identification and quantification of saliva miRNA and microbial RNA was performed using next generation sequencing (NGS) on a NextSeq 500 instrument (Illumina), following the TruSeq Small RNA Library Preparation Kit protocol (Illumina, San Diego, CA). Alignment of mature miRNA reads was performed with the miRbase21 database using the Shrimp2 algorithm in Partek Flow software (Partek, Inc., St. Louis, MO). Mapping of unique microbial transcripts was performed using the K-Slam database, which references the NCBI Taxonomy database [46]. Taxons were defined by their family, genus, species, and subspecies (when available). The human miRNAs and microbial RNAs present in raw counts of 10 or more in at least 10% of samples were interrogated for oscillating expression. A quantile normalization technique was applied to the human miRNA and microbial RNA datasets separately, prior to statistical analysis.

### Identification of oscillating salivary RNAs

A two-way analysis of variance (ANOVA) was performed using sample sets 1 and 2 based on binning the samples into their approximately replicated collection times, to identify host miRNAs and microbial RNAs that varied significantly (FDR<0.05) with collection time but not the day of collection (in order to eliminate RNAs which could be influenced by daily variations in routine). A subset of miRNAs and microbial RNAs that were highly associated with time of collection (R ≥ 0.90 or 0.84 in sample sets 1 and 2, respectively; p<0.001) were then used in a naïve hold-out set (sample set 3) to assess predictive accuracy for time of collection with a multivariate regression analysis. The miRNAs that showed the strongest circadian oscillations were termed *CircaMiRs* and the microbes that displayed the strongest oscillations in transcriptional activity were termed *CircaMicrobes*. Relationships between CircaMiRs and CircaMicrobes were investigated with a Pearson Correlation analysis.

### Functional Interrogation of CircaMiRs and CircaMicrobes

Classification of the mapped microbial RNAs within defined metabolic and functional categories was performed through conversion of microbial reads to Kyoto Encyclopedia of Genes and Genomes (KEGG) Orthology identifiers (KO IDs) which were mapped by MicrobiomeAnalyst software[47] to a set of 202 different KEGG Modules, KEGG Pathways, and COG Categories. For each participant, KEGG and COG data were summed across four collection periods (i.e. 7–9 AM, 10 AM–2 PM, 3–6 PM, 7–10 PM) for all of the days saliva samples were collected. Changes in expression of individual functional clusters were explored with a non-parametric Kruskal Wallis ANOVA. Patterns in functional clusters across the four time periods were visualized with hierarchical clustering analysis and a partial least squares discriminant analysis in MetaboAnalyst software.

The potential biologic impact of the CircaMiRs was investigated through functional annotation of their high confidence mRNA targets (p<0.05, Micro-T Score ≥ 0.95) in DIANA miRPath v3 software and Ingenuity Pathway Analyst software (IPA, Qiagen). KEGG pathways over-represented by these mRNA targets were determined with Fisher’s Exact test with FDR correction (FDR<0.05). Inter-relatedness of protein products for the mRNA targets was explored in String v10.5. Alignment of salivary RNA to the RefSeq Transcripts database in Partek Flow permitted quantification of local (oropharyngeal) mRNA targets for salivary CircaMiRs (that were ≤ 50 base pairs). Relationships between CircaMiRs and mRNA targets were explored with Pearson’s correlations.

To further explore the potential biological significance of the miRNA data, we examined the levels of the oscillating salivary CircaMiRs in the same cohort of 2–6 year old children with ASD examined for miRNA expression who either had normal sleep patterns (n = 63) or disordered sleep symptoms (n = 77). Group differences in mean salivary CircaMiR expression between the sleep disorder and non-sleep disorder groups were identified with a non-parametric Mann Whitney U-test. A two-way ANOVA assessed relationships between CircaMiRs, disordered sleep, and collection time, as well as sleep disorder-time interactions. Finally, a multivariate linear regression was used to determine the ability of the most robust CircaMiRs to predict collection time in the ASD children with and without sleep disorders.

### Influence of daily routines on the oral transcriptome

To investigate the potential impact of daily routines on salivary miRNA and microbial RNA levels we examined associations between the oral transcriptome in sample set 3 and: 1) time since last meal (in hours); 2) time since last tooth brushing (in hours); and 3) time since last sleep (in hours). Significant relationships (|R|>0.40; FDR<0.05) between these three variables and salivary RNA levels were reported.

## Supporting information

S1 Table Circadian miRNAs in sample sets 1 and 2 identified by two-way ANOVA. (XLSX)

S2 Table Gene targets of Circadian miRNAs in sample sets 1 and 2. (XLSX)

S3 Table Pathways enriched in CircaMiR target genes. (XLSX)

S4 Table Associations of CircaMiRs with disordered sleep in ASD children. (XLSX)

## References

[1] Takeda N, Maemura K. Circadian clock and the onset of cardiovascular events. Hypertension Research. 2016;39:383 10.1038/hr.2016.9 26888119

[2] Reinke H, Asher G. Circadian Clock Control of Liver Metabolic Functions. Gastroenterology. 2016;150(3):574–80. Epub 2015/12/15. 10.1053/j.gastro.2015.11.043 . 26657326

[3] Videnovic A, Zee PC. Consequences of Circadian Disruption on Neurologic Health. Sleep medicine clinics. 2015;10(4):469–80. Epub 2015/11/17. 10.1016/j.jsmc.2015.08.004 ; PubMed Central PMCID: PMCPMC4648713. 26568123 PMC4648713

[4] Buhr ED, Takahashi JS. Molecular components of the Mammalian circadian clock. Handbook of experimental pharmacology. 2013;(217):3–27. Epub 2013/04/23. 10.1007/978-3-642-25950-0_1 ; PubMed Central PMCID: PMCPMC3762864. 23604473 PMC3762864

[5] Stoicea N, Du A, Lakis DC, Tipton C, Arias-Morales CE, Bergese SD. The MiRNA Journey from Theory to Practice as a CNS Biomarker. Frontiers in genetics. 2016;7:11 Epub 2016/02/24. 10.3389/fgene.2016.00011 ; PubMed Central PMCID: PMCPMC4746307. 26904099 PMC4746307

[6] Guan Y, Mizoguchi M, Yoshimoto K, Hata N, Shono T, Suzuki SO, et al MiRNA-196 is upregulated in glioblastoma but not in anaplastic astrocytoma and has prognostic significance. Clinical cancer research: an official journal of the American Association for Cancer Research. 2010;16(16):4289–97. Epub 2010/07/06. 10.1158/1078-0432.ccr-10-0207 . 20601442

[7] Gallego JA, Gordon ML, Claycomb K, Bhatt M, Lencz T, Malhotra AK. In vivo microRNA detection and quantitation in cerebrospinal fluid. Journal of molecular neuroscience: MN. 2012;47(2):243–8. Epub 2012/03/10. 10.1007/s12031-012-9731-7 ; PubMed Central PMCID: PMCPMC3361591. 22402993 PMC3361591

[8] Cheng L, Quek CY, Sun X, Bellingham SA, Hill AF. The detection of microRNA associated with Alzheimer's disease in biological fluids using next-generation sequencing technologies. Frontiers in genetics. 2013;4:150 Epub 2013/08/22. 10.3389/fgene.2013.00150 ; PubMed Central PMCID: PMCPMC3737441. 23964286 PMC3737441

[9] Freischmidt A, Muller K, Ludolph AC, Weishaupt JH. Systemic dysregulation of TDP-43 binding microRNAs in amyotrophic lateral sclerosis. Acta neuropathologica communications. 2013;1:42 Epub 2013/11/21. 10.1186/2051-5960-1-42 ; PubMed Central PMCID: PMCPMC3893596. 24252274 PMC3893596

[10] Siegel SR, Mackenzie J, Chaplin G, Jablonski NG, Griffiths L. Circulating microRNAs involved in multiple sclerosis. Molecular biology reports. 2012;39(5):6219–25. Epub 2012/01/11. 10.1007/s11033-011-1441-7 . 22231906

[11] Ksiazek-Winiarek DJ, Kacperska MJ, Glabinski A. MicroRNAs as novel regulators of neuroinflammation. Mediators of inflammation. 2013;2013:172351 Epub 2013/08/29. 10.1155/2013/172351 ; PubMed Central PMCID: PMCPMC3745967. 23983402 PMC3745967

[12] Sheinerman KS, Umansky SR. Circulating cell-free microRNA as biomarkers for screening, diagnosis and monitoring of neurodegenerative diseases and other neurologic pathologies. Front Cell Neurosci. 2013;7:150 Epub 2013/09/24. 10.3389/fncel.2013.00150 ; PubMed Central PMCID: PMCPMC3767917. 24058335 PMC3767917

[13] Cloutier F, Marrero A, O'Connell C, Morin P Jr., MicroRNAs as potential circulating biomarkers for amyotrophic lateral sclerosis. Journal of molecular neuroscience: MN. 2015;56(1):102–12. Epub 2014/12/01. 10.1007/s12031-014-0471-8 . 25433762

[14] Galimberti D, Villa C, Fenoglio C, Serpente M, Ghezzi L, Cioffi SM, et al Circulating miRNAs as potential biomarkers in Alzheimer's disease. Journal of Alzheimer's disease: JAD. 2014;42(4):1261–7. Epub 2014/07/16. 10.3233/JAD-140756 . 25024331

[15] Hansen KF, Sakamoto K, Obrietan K. MicroRNAs: a potential interface between the circadian clock and human health. Genome Med. 2011;3(2):10 Epub 2011/02/25. 10.1186/gm224 ; PubMed Central PMCID: PMCPMC3092095. 21345247 PMC3092095

[16] Weber JA, Baxter DH, Zhang S, Huang DY, Huang KH, Lee MJ, et al The microRNA spectrum in 12 body fluids. Clinical chemistry. 2010;56(11):1733–41. Epub 2010/09/18. 10.1373/clinchem.2010.147405 ; PubMed Central PMCID: PMCPMC4846276. 20847327 PMC4846276

[17] de Kloet ER, Fitzsimons CP, Datson NA, Meijer OC, Vreugdenhil E. Glucocorticoid signaling and stress-related limbic susceptibility pathway: about receptors, transcription machinery and microRNA. Brain Res. 2009;1293:129–41. Epub 2009/04/01. 10.1016/j.brainres.2009.03.039 . 19332027

[18] Wang Y, Mead EA, Thekkumthala AP, Pietrzykowski AZ. New Players in the Neuroendocrine System: A Journey Through the Non-coding RNA World Molecular Neuroendocrinology: From Genome to Physiology: John Wiley & Sons, Ltd; 2015 p. 75–96.

[19] Cheng H-YM, Papp JW, Varlamova O, Dziema H, Russell B, Curfman JP, et al microRNA Modulation of Circadian-Clock Period and Entrainment. Neuron. 2007;54(5):813–29. 10.1016/j.neuron.2007.05.017. 10.1016/j.neuron.2007.05.017 17553428 PMC2590749

[20] Masotti A. Interplays between gut microbiota and gene expression regulation by miRNAs. Frontiers in Cellular and Infection Microbiology. 2012;2:137 10.3389/fcimb.2012.00137. PMC3487149. 23130352 PMC3487149

[21] Dalmasso G, Nguyen HT, Yan Y, Laroui H, Charania MA, Ayyadurai S, et al Microbiota modulate host gene expression via microRNAs. PLoS One. 2011;6(4):e19293 Epub 2011/05/12. 10.1371/journal.pone.0019293 ; PubMed Central PMCID: PMCPMC3084815. 21559394 PMC3084815

[22] O'Mahony SM, Clarke G, Borre YE, Dinan TG, Cryan JF. Serotonin, tryptophan metabolism and the brain-gut-microbiome axis. Behav Brain Res. 2015;277:32–48. Epub 2014/08/01. 10.1016/j.bbr.2014.07.027 . 25078296

[23] Dickson I. Gut microbiota: Intestinal microbiota oscillations regulate host circadian physiology. Nature Reviews Gastroenterology & Hepatology. 2017;14(67). 10.1038/nrgastro.2016.205 27999435

[24] Heegaard NH, Carlsen AL, Lilje B, Ng KL, Ronne ME, Jorgensen HL, et al Diurnal Variations of Human Circulating Cell-Free Micro-RNA. PLoS One. 2016;11(8):e0160577 Epub 2016/08/06. 10.1371/journal.pone.0160577 ; PubMed Central PMCID: PMCPMC4975411. 27494182 PMC4975411

[25] Gorostiza EA, Depetris-Chauvin A, Frenkel L, Pírez N, Ceriani María F. Circadian Pacemaker Neurons Change Synaptic Contacts across the Day. Current Biology. 2014;24(18):2161–7. 10.1016/j.cub.2014.07.063. 10.1016/j.cub.2014.07.063 25155512 PMC4175170

[26] Jasinska M, Grzegorczyk A, Woznicka O, Jasek E, Kossut M, Barbacka-Surowiak G, et al Circadian rhythmicity of synapses in mouse somatosensory cortex. European Journal of Neuroscience. 2015;42(8):2585–94. 10.1111/ejn.13045 26274013

[27] Jasinska M, Pyza E. Circadian Plasticity of Mammalian Inhibitory Interneurons. Neural plasticity. 2017;2017:6373412 Epub 2017/04/04. 10.1155/2017/6373412 ; PubMed Central PMCID: PMCPMC5358450 publication of this paper. 28367335 PMC5358450

[28] Damdimopoulou P, Nurmi T, Salminen A, Damdimopoulos AE, Kotka M, van der Saag P, et al A single dose of enterolactone activates estrogen signaling and regulates expression of circadian clock genes in mice. The Journal of nutrition. 2011;141(9):1583–9. Epub 2011/07/15. 10.3945/jn.111.140277 . 21753063

[29] Laudes M, Oberhauser F, Schulte DM, Freude S, Bilkovski R, Mauer J, et al Visfatin/PBEF/Nampt and resistin expressions in circulating blood monocytes are differentially related to obesity and type 2 diabetes in humans. Hormone and metabolic research = Hormon- und Stoffwechselforschung = Hormones et metabolisme. 2010;42(4):268–73. Epub 2010/01/22. 10.1055/s-0029-1243638 . 20091460

[30] Figueroa KP, Farooqi S, Harrup K, Frank J, O'Rahilly S, Pulst SM. Genetic variance in the spinocerebellar ataxia type 2 (ATXN2) gene in children with severe early onset obesity. PLoS One. 2009;4(12):e8280 Epub 2009/12/18. 10.1371/journal.pone.0008280 ; PubMed Central PMCID: PMCPMC2791421. 20016785 PMC2791421

[31] Hanson JR. Terpenoids and steroids Royal Society of Chemistry; 2007.

[32] Son GH, Chung S, Choe HK, Kim HD, Baik SM, Lee H, et al Adrenal peripheral clock controls the autonomous circadian rhythm of glucocorticoid by causing rhythmic steroid production. Proc Natl Acad Sci U S A. 2008;105(52):20970–5. Epub 2008/12/19. 10.1073/pnas.0806962106 ; PubMed Central PMCID: PMCPMC2634940. 19091946 PMC2634940

[33] Witwer KW. Circulating microRNA biomarker studies: pitfalls and potential solutions. Clinical chemistry. 2015;61(1):56–63. Epub 2014/11/14. 10.1373/clinchem.2014.221341 . 25391989

[34] Hicks SD, Middleton FA. A Comparative Review of microRNA Expression Patterns in Autism Spectrum Disorder. Front Psychiatry. 2016;7:176 Epub 2016/11/22. 10.3389/fpsyt.2016.00176 ; PubMed Central PMCID: PMCPMC5095455. 27867363 PMC5095455

[35] Liu S, da Cunha AP, Rezende RM, Cialic R, Wei Z, Bry L, et al The Host Shapes the Gut Microbiota via Fecal MicroRNA. Cell host & microbe. 2016;19(1):32–43. Epub 2016/01/15. 10.1016/j.chom.2015.12.005 ; PubMed Central PMCID: PMCPMC4847146. 26764595 PMC4847146

[36] Klingelhoefer L, Reichmann H. Pathogenesis of Parkinson disease—the gut-brain axis and environmental factors. Nat Rev Neurol. 2015;11(11):625–36. Epub 2015/10/28. 10.1038/nrneurol.2015.197 . 26503923

[37] Li Q, Zhou JM. The microbiota–gut–brain axis and its potential therapeutic role in autism spectrum disorder. Neuroscience. 2016;324(Supplement C):131–9. 10.1016/j.neuroscience.2016.03.013. 26964681

[38] Carskadon MA, Wolfson AR, Acebo C, Tzischinsky O, Seifer R. Adolescent sleep patterns, circadian timing, and sleepiness at a transition to early school days. Sleep. 1998;21(8):871–81. Epub 1999/01/01. . 9871949 10.1093/sleep/21.8.871

[39] Wulff K, Gatti S, Wettstein JG, Foster RG. Sleep and circadian rhythm disruption in psychiatric and neurodegenerative disease. Nat Rev Neurosci. 2010;11(8):589–99. Epub 2010/07/16. 10.1038/nrn2868 . 20631712

[40] Hicks SD, Ignacio C, Gentile K, Middleton FA. Salivary miRNA profiles identify children with autism spectrum disorder, correlate with adaptive behavior, and implicate ASD candidate genes involved in neurodevelopment. BMC Pediatr. 2016;16:52 Epub 2016/04/24. 10.1186/s12887-016-0586-x ; PubMed Central PMCID: PMCPMC4841962. 27105825 PMC4841962

[41] Hicks SD, Johnson J, Carney MC, Bramley H, Olympia RP, Loeffert AC, et al Overlapping MicroRNA Expression in Saliva and Cerebrospinal Fluid Accurately Identifies Pediatric Traumatic Brain Injury. Journal of neurotrauma. 2017 Epub 2017/08/02. 10.1089/neu.2017.5111 . 28762893 PMC7227420

[42] Ainsworth D, Sternberg MJE, Raczy C, Butcher SA. k-SLAM: accurate and ultra-fast taxonomic classification and gene identification for large metagenomic data sets. Nucleic Acids Res. 2017;45(4):1649–56. Epub 2016/12/15. 10.1093/nar/gkw1248 ; PubMed Central PMCID: PMCPMC5389551. 27965413 PMC5389551

[43] Dhariwal A, Chong J, Habib S, King IL, Agellon LB, Xia J. MicrobiomeAnalyst: a web-based tool for comprehensive statistical, visual and meta-analysis of microbiome data. Nucleic Acids Res. 2017 Epub 2017/04/28. 10.1093/nar/gkx295 ; PubMed Central PMCID: PMCPMC5570177. 28449106 PMC5570177

[44] Ramsey KM, Yoshino J, Brace CS, Abrassart D, Kobayashi Y, Marcheva B, et al Circadian clock feedback cycle through NAMPT-mediated NAD+ biosynthesis. Science. 2009;324(5927):651–4. Epub 2009/03/21. 10.1126/science.1171641 ; PubMed Central PMCID: PMCPMC2738420. 19299583 PMC2738420

[45] Bendova Z, Sladek M, Svobodova I. The expression of NR2B subunit of NMDA receptor in the suprachiasmatic nucleus of Wistar rats and its role in glutamate-induced CREB and ERK1/2 phosphorylation. Neurochemistry international. 2012;61(1):43–7. Epub 2012/05/01. 10.1016/j.neuint.2012.04.016 . 22543102

[46] Wang WX, Huang Q, Hu Y, Stromberg AJ, Nelson PT. Patterns of microRNA expression in normal and early Alzheimer's disease human temporal cortex: white matter versus gray matter. Acta neuropathologica. 2011;121(2):193–205. Epub 2010/10/12. 10.1007/s00401-010-0756-0 ; PubMed Central PMCID: PMCPMC3073518. 20936480 PMC3073518

[47] Song J, Kim YK. Identification of the Role of miR-142-5p in Alzheimer's Disease by Comparative Bioinformatics and Cellular Analysis. Frontiers in molecular neuroscience. 2017;10:227 Epub 2017/08/05. 10.3389/fnmol.2017.00227 ; PubMed Central PMCID: PMCPMC5513939. 28769761 PMC5513939

